# Fatal Outbreak from Consuming *Xanthium strumarium* Seedlings during Time of Food Scarcity in Northeastern Bangladesh

**DOI:** 10.1371/journal.pone.0009756

**Published:** 2010-03-18

**Authors:** Emily S. Gurley, Mahmudur Rahman, M. Jahangir Hossain, Nazmun Nahar, M. Abul Faiz, Nazrul Islam, Rebeca Sultana, Selina Khatun, Mohammad Zashim Uddin, M. Sabbir Haider, M. Saiful Islam, Be-Nazir Ahmed, Muhammad Waliur Rahman, Utpal Kumar Mondal, Stephen P. Luby

**Affiliations:** 1 Programme on Infectious Diseases and Vaccine Sciences, International Centre for Diarrheal Diseases Research, Bangladesh (ICDDR,B), Dhaka, Bangladesh; 2 Institute of Epidemiology, Disease Control and Research (IEDCR), Ministry of Health and Family Welfare, Government of Bangladesh, Dhaka, Bangladesh; 3 Directorate General of Health Services, Ministry of Health and Family Welfare, Government of Bangladesh, Dhaka, Bangladesh; 4 Department of Virology, Bangabandhu Sheikh Mujib Medical University, Dhaka, Bangladesh; 5 Department of Botany, University of Dhaka, Dhaka, Bangladesh; 6 Centers for Disease Control and Prevention, Atlanta, Georgia, United States of America; Aga Khan University, Pakistan

## Abstract

**Background:**

An outbreak characterized by vomiting and rapid progression to unconsciousness and death was reported in Sylhet Distrct in northeastern Bangladesh following destructive monsoon floods in November 2007.

**Methods and Findings:**

We identified cases presenting to local hospitals and described their clinical signs and symptoms. We interviewed patients and their families to collect illness histories and generate hypotheses about exposures associated with disease. An epidemiological study was conducted in two outbreak villages to investigate risk factors for developing illness. 76 patients were identified from 9 villages; 25% (19/76) died. Common presenting symptoms included vomiting, elevated liver enzymes, and altered mental status. In-depth interviews with 33 cases revealed that 31 (94%) had consumed *ghagra shak*, an uncultivated plant, in the hours before illness onset. *Ghagra shak* was consumed as a main meal by villagers due to inaccessibility of other foods following destructive monsoon flooding and rises in global food prices. Persons who ate this plant were 34.2 times more likely (95% CI 10.2 to 115.8, p-value<0.000) than others to develop vomiting and unconsciousness during the outbreak in our multivariate model. *Ghagra shak* is the local name for *Xanthium strumarium*, or common cocklebur.

**Conclusions:**

The consumption of *Xanthium strumarium* seedlings in large quantities, due to inaccessibility of other foods, caused this outbreak. The toxic chemical in the plant, carboxyatratyloside, has been previously described and eating *X. strumarium* seeds and seedlings has been associated with fatalities in humans and livestock. Unless people are able to meet their nutritional requirements with safe foods, they will continue to be at risk for poor health outcomes beyond undernutrition.

## Introduction

Food insecurity is a chronic problem in Bangladesh, but food became increasingly scarce during 2007. Bangladesh relies upon yearly monsoon floods to produce its rice crops but during July–September 2007 the floods became destructive and approximately 13% of the *aman* rice crop was destroyed [1]. This crop loss was accompanied by rises in global food prices [2] and between October 2006 and October 2007 the price of rice, which is eaten by most Bangladeshi households at every meal, increased by 38% [1]. As a result, between 2005 and 2008, the number of people in Bangladesh who received less than 1805 calories per day increased by 6.8 million and in 2008 affected approximately 24% of the population [1].

On 4 November 2007, following the destructive monsoon floods, a woman and her child presented unconscious to a government primary care hospital in Sylhet District in northeastern Bangladesh. Relatives explained that the patients had experienced brief episodes of vomiting and restlessness prior to losing consciousness and within hours, the woman and child died. In the following days, 18 patients with similar symptoms from two sub-districts presented to local hospitals. An investigation of this outbreak, jointly undertaken by the Institute of Epidemiology, Disease Control and Research (IEDCR), Ministry of Health and Family Welfare, Government of Bangladesh and the International Centre for Diarrheal Disease Research, Bangladesh (ICDDR,B), began on 5 November 2007. The objectives of the investigation were to determine the etiology of disease, risk factors for developing disease, and to develop prevention strategies.

## Methods

### Case detection and clinical investigation

Based on presenting symptoms of the index case, we defined a probable case as anyone who had vomiting and altered mental status (including disorientation, extreme lethargy, or unconsciousness) with onset on or after 2 November 2007 and a suspect case as someone who experienced vomiting without altered mental status during the same time period who resided in the same village as a probable case and had illness onset within 3 days of the probable case. Probable and suspect cases were identified by screening all patients presenting to local government health facilities in the affected areas. Cases occurring in the community which were reported to the study team by hospitalized cases or their caregivers were also included in the clinical line list. Many of these cases had died before seeking care at a health facility.

We recorded clinical histories for both probable and suspect cases using a structured questionnaire. Blood was collected from hospitalized cases and, when possible, serological and liver function tests performed. Magnetic resonance imaging (MRI) of the brain and cerebrospinal fluid (CSF) analysis were conducted on a sub-set of patients presenting to hospital with altered mental status. Pathological autopsies are not routinely performed in Bangladesh and, therefore, histological examinations were not done. Tests for infectious diseases known to cause severe disease in Bangladesh, including influenza A, Nipah virus, Japanese encephalitis, and malaria, were performed on a subset of case-patients.

### Anthropological investigation

**Figure 1:**
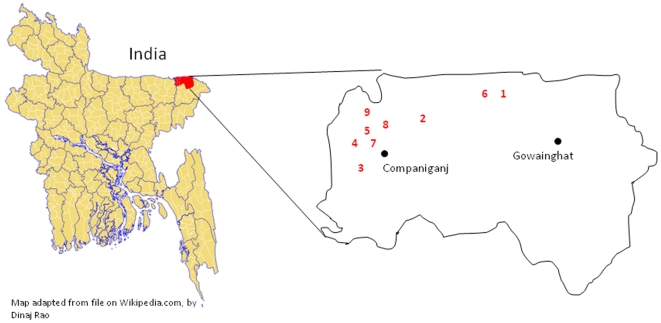
Approximate location of outbreak villages numbered to indicate order in which each village became affected by the outbreak.

Anthropologists trained in outbreak investigation visited hospitalized patients to collect detailed illness histories and investigate common exposures among cases, including exposure to sick animals and humans, and travel and food histories, in order to generate hypotheses about the cause of the outbreak which could be tested in an epidemiological study. Due to the rapid onset and progression of illness, we suspected that the outbreak was caused by a toxic exposure. They visited the 4 villages first identified during the outbreak (Figure 1), and conducted in-depth and group interviews with families and neighbors of cases to describe in detail the activities of cases in the 24 hours prior to developing illness. The team questioned cases and their families about possible man-made chemical exposures, and listed any and all goods which had been purchased or brought into affected households during the week prior to the outbreak. The team also enquired about all foods consumed, purchased, or cooked by case households in the day before illness onset. They also probed about consumption of uncultivated plants based on evidence from India where outbreaks with similar clinical characteristics were caused by consuming toxic wild plants [3]–[5].

### Epidemiological study

The villages involved in the outbreak were remote and travel was made more difficult as the floods had damaged local roads and bridges. Therefore, given time and logistical constraints, we chose to conduct the epidemiologic study in 2 villages, including the index village, which represented 41% (31/76) of all cases, 51% (23/45) of all probable cases, and 58% of identified deaths (11/19) (Figure 1, villages 1 and 3). Trained data collectors went door-to-door in these villages to collect information from all residents about exposures and illnesses including vomiting during the outbreak period. We asked respondents about their exposure to sick animals and to report the foods that they consumed during the outbreak period. We defined suspect illness as vomiting and probable illness as vomiting with unconsciousness in the two days before the first death through the day the last death occurred in the village. We used unconsciousness in the probable case definition in the community survey, rather than altered mental status as was used in the clinical case finding activity, because we believed that unconsciousness would be more reliably reported by persons without medical training.

All persons who were living in the two selected villages during the outbreak were asked to participate in the study. Appropriate proxy respondents, persons who could reasonably report on the subject's exposures, were identified for children and persons hospitalized or deceased.

We estimated the association between exposures and illness by calculating odds ratios with exact 95% confidence intervals and considered results with a p-value ≤0.05 to be statistically significant. We conducted 2 separate analyses using both the suspect and probable case definitions. If no cases reported an exposure, we did not calculate confidence intervals or p-values for the odds ratio. In our multivariate analysis, we used generalized estimating equations to account for the clustering of exposures, particularly of foods consumed, observed in households [6]. All exposures with associated p-values of 0.2 or less in the univariate analysis were included in the multivariate model.

### Environmental study

We collected specimens of uncultivated plans consumed by villagers and sent them to Dhaka University where the species was determined by local botanists.

### Ethics

This study was conducted at the request of the Government of Bangladesh to inform public health prevention strategies to respond to the outbreak. Because it was an outbreak investigation, the study protocol was not reviewed by a human subjects committee. However, the study was approved by the Government of Bangladesh and informed, verbal consent was sought from all respondents or their proxies and guardians. Verbal consent was deemed appropriate for this setting because our investigations posed no more than minimal risk to participants and would not require written consent outside of the research context.

## Results

### Description of cases and clinical syndrome

**Figure 2:**
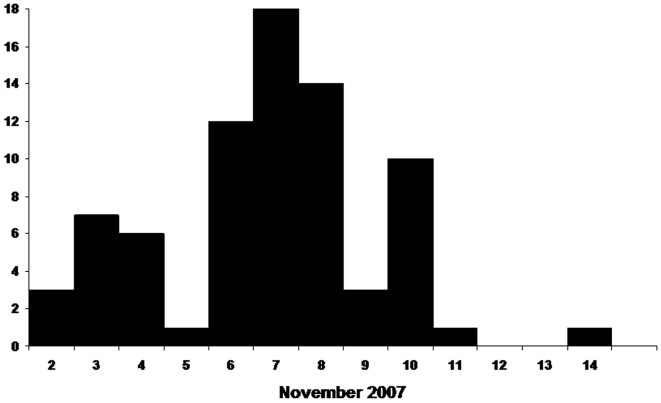
Dates of onset of vomiting in patients identified during the outbreak, n = 76.

There were 76 cases identified from 9 villages in remote areas of Sylhet District within 15 km of the border with India. (Figure 1) Fifty-four of these cases presented for treatment at local hospitals and 22 were reported to investigators by hospitalized cases or their families. All cases occurred over a 13 day period from November 2–14. (Figure 2) Cases were predominantly female (70%) and the mean age was 16.8 years. The majority of cases reported vomiting (100%), history of fever (61%), and altered mental status (59%); the case fatality ratio was 25% (19/76) (Table 1). Onset of mild fever was commonly reported by case-patients after vomiting began, however, of the 63 case-patients who had their temperature taken during their illness only 21% (16/63) had an elevated temperature between 99 and102°F (median 99.8 °F). Eighty-four percent (16/19) of deaths were in children ≤15 years old; the case fatality ratio for children aged ≤5 years was 33%, was 36% for children older than 5 and ≤10, but was only 10% for those >15 years of age.

**Table 1:** Demographic characteristics and clinical description of suspect and probable cases (N = 76).

Age in completed years (mean, range)		16.8,1–55
		n(%)
Female		53(70)
Clinical signs and symptoms		
Vomiting*		76(100)
History of fever		46(61)
Altered mental status		45(59)
Unconsciousness**		29(38)
Fatigue/drowsiness		19(25)
Diarrhoea		9(12)
Difficulty breathing		12(16)
Irritability		10(13)
Blood in vomitus		9(12)
Headache		8(11)
Frothy discharge from mouth		8(11)
Died		19(25)
Laboratory testing***	n	mean; range
White blood cells	19	
Total count/cmm		13,074; 5900–25,000
Polymorphs (%)		73; 44–90
Lymphocytes (%)		20; 6–36
Platelets/cmm	17	263,000; 130,000–420,000
Serum electrolytes (µmol/L)	19	
Sodium		145; 129–149
Potassium		4.2; 3–5.5
Chloride		108; 90–116
Carbon dioxide		27; 18–37
Haemaglobin (gm/dl)	17	11.3; 9.1–13.1
Prothrombin time (seconds)	17	27; 12–69
Aspartate aminotransferase (U/L)	5	174; 111–336
Serum bilirubin (µmol/L)	19	1.2; 0.4–5.1
Alanine aminotransferase (U/L)	23	1871; 15–10,000

*Per the case definition.

**Those with unconsciousness are a sub-set of those with altered mental status.

***Laboratory testing was not performed for all cases; the number of cases tested is indicated by n.

Laboratory investigations were performed on a sub-set of hospitalized case-patients. Ten patients received diagnostic testing for malaria, Japanese encephalitis, Nipah virus, and influenza A; all tests were all negative. One patient presenting with altered mental status had an MRI of the brain and an examination of cerebrospinal fluid. Both were normal. Patients had laboratory evidence of liver damage, evidenced by elevated alanine aminotransferase (ALT) and serum bilirubin levels and increased prothrombin times (Table 1). Ten of 17 patients who had ALT levels tested had levels >6 times the normal value of 40 U/L. Nine of the 29 cases with unconsciousness (21%) and 6 of the 19 deaths (19%) were never seen by physicians.

### Anthropological investigation

Outbreak villages were located in remote areas, up to a 3 km walk from the nearest paved road. Case households reported that during the weeks preceding the outbreak they were unable to meet their daily food needs and typically ate less than three meals per day. The household wage earners were day laborers engaged primarily in collecting stones, fishing, and moving earth for construction projects. Recent prohibitions on collecting stones from nearby rivers had decreased local income and in the month before the outbreak most of their poultry died from disease which put additional constraints on available food resources. Residents rarely traveled outside of their village.

Villagers reported that cases became ill suddenly with frequent and profuse vomiting, followed quickly (within minutes or hours) by unconsciousness for some. In the index household, a mother and her 3 children became ill within 6 hours of each other and all but 1 child died within 12 hours of onset of illness.

A primary goal of the qualitative investigation was to determine if cases shared a common exposure prior to illness. In total, the qualitative team collected in-depth exposure histories for 33 cases from the first 4 villages identified during the outbreak (Figure 1), including 14 who died. We could identify no common environmental or chemical exposures among cases. However, of the 33 cases investigated, 31 reportedly ate *ghagra shak*, an uncultivated plant, within 24 hours before they became ill. All cases also reportedly consumed small fish, collected from nearby ponds, in the 24 hours before onset of illness. In addition to *ghagra shak*, villagers reported eating 4 other kinds of uncultivated plants in the weeks before the outbreak. They reported that they relied upon uncultivated plants and fish more than usual due to recent crop destruction. They reported that food in the markets was prohibitively expensive because of the local food shortage.

Locals described their usual *ghagra shak* collection and preparation practices for the team. The plant grows nearby their homes and they commonly use the leaves and stalks of mature plants to flavor curries or for medicinal purposes although they typically don not eat *ghagra shak* seedlings. In the absence of other food sources this year, however, village residents reported that their *ghagra shak* consumption pattern changed. They ate *ghagra shak* as a main meal and they had consumed immature plants as they sprouted. As the father of a child who died explained:

*“Now we are facing (food) scarcity; this year's scarcity is severe. Although we eat three times a day, the amount (we eat) is small. We need 200 taka (approximately US$2.85) for our daily requirements but we cannot manage it. This year's flooding has created a problem for us. Now* ghagra shak *will grow and we will eat* ghagra shak.*”*

Villagers and local officials suspected that consuming *ghagra shak* was responsible for the outbreak. In households where family members died, and particularly in the index village, survivors were reluctant to admit to serving *ghagra shak* to avoid blame for the deaths.

### Epidemiological study

One hundred thirty-one households were surveyed; 647 persons were interviewed (85% of all residents). Individuals not interviewed were unable to be contacted on multiple visits to the household. Sixty-seven percent of households (88/131) lived off of less than $2 per day.

Fifty-six persons reported experiencing vomiting during the outbreak period and 26 experienced both vomiting and unconsciousness. During our case finding in hospitals, we only identified 15 cases with vomiting and unconsciousness from these villages; 11 additional cases were identified during this house-to-house survey. The results of our statistical analyses using two different case definitions were similar and we present here in detail the results from the analysis using vomiting with unconsciousness as the case definition. Persons who reported eating *ghagra shak* during the outbreak period were 21.6 times more likely (95% confidence internal, 8.1–55.9, p-value<0.000) to experience vomiting and unconsciousness than those who did not eat the plant in univariate analysis. (Table 2) Statistically significant risk of illness was also associated with consuming cucumber, lentils (both *kheshari*, the least expensive type of lentils available, and *mashoor*, a more expensive variety), day-old rice with water (called *panta bhat*, this is sometimes fermented), string beans, and red spinach. When household clustering was controlled for in the multivariate model the odds of developing illness after eating *gaghra shak* was 34.2 (95% CI 10.1–115.8, p-value<0.000) and for *keshari* lentils was 9.1 (95% CI 1.3–65.2, p-value = 0.028). However, eating *ghagra shak* explained the largest proportion of cases with vomiting and unconsciousness (46%). (Table 2) In the multivariate analysis using vomiting as the case definition, the odds ratio associated the with eating *ghagra shak* was 8.0 (95% CI 3.2–20.1, p<0.000) and *keshari* lentils was 5.5 (95% CI 1.2–26.0, p-value = 0.03).

**Table 2:** Risk of experiencing vomiting with unconsciousness (N = 26) in two villages during the outbreak period by type of food consumed.

Foods									
	Ate			Did not eat					
	# Cases	Total	Attack rate %	# Cases	Total	Attack rate %	Odds ratio	95% Confidence interval	P-value
*Mashoor* lentils (smaller, more expensive)	7	301	2	19	353	5	0.4	0.2–1.1	0.046*
*Kheshari* lentils (larger, the least expensive available)	5	13	38	21	641	3	18.5	4.3–69.7	<0.001*
Day-old rice with water, sometimes fermented (*panta bhat*)	3	20	15	23	634	4	4.7	0.8–17.9	0.011*
Wheat	0	17	0	26	637	4	0	-	-
Dried fish	8	180	4	18	474	4	1.2	0.4–2.9	0.705
Potato	7	197	4	19	457	4	0.9	0.3–2.2	0.717
Banana	3	41	12	23	613	4	2.0	0.4–7.2	0.258
Cucumber	2	9	22	24	645	4	7.4	0.7–41	0.005*
String beans (*borboti*)	7	62	11	19	592	3	3.8	1.3–10	0.002*
Red spinach	15	230	7	11	424	3	2.6	1.1–6.4	0.014*
Radish leaves	10	234	4	16	420	4	1.1	0.5–2.7	0.771
Chicken	0	43	0	26	611	4	0	-	-
Egg	2	67	3	24	587	4	0.7	0.1–3.0	0.661
Beef	0	4	0	26	650	4	0	-	-
Other meat	0	5	0	26	649	4	0	-	-
Uncultivated plants									
*Gaghra shak* (*Xanthium strumarium*)	12	36	33	14	618	2	21.6	8.1–55.9	<0.000*
*Getchu* root (*Apanogeton natans*)	3	29	10	23	625	4	3.0	0.6–11.0	0.073
*Haincha shak* (*Alternanthera sessilis*)	5	120	4	21	534	4	1.1	0.3–3.0	0.906
Ferns	2	17	12	24	637	4	3.4	0.4–16.0	0.096
Root of aram (*Colocasia esculenta*)	1	53	2	25	601	4	0.4	0.01–2.8	0.417
Small freshwater fish									
*Gutum* (*Lepidocephalus annandalei* or *guntea*)	13	238	5	13	416	3	1.8	0.8–4.3	0.141
Kailsha (*Colisa fasciatus*)	8	211	4	18	443	4	0.9	0.3–2.3	0.868
*Punti* (*Puntius puntio*)	16	419	4	10	235	4	0.9	0.4–2.2	0.784
*Baim* (*Mastacembelus armatus*)	7	154	5	19	500	4	1.2	0.4–3.1	0.679
*Potka* (A kind of puffer fish, species unknown)	0	12	0	26	642	4	0	-	-

*P-value statistically significant ≤0.05.

### Environmental study

**Figure 3:**
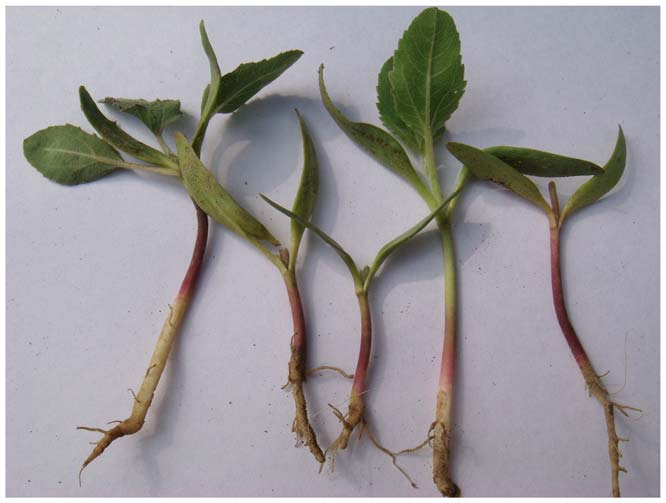
*Ghagra shak* (*Xanthium strumarium*) plants picked from the index village during the outbreak.

Local botanists determined that the plant known to villagers as *ghagra shak* was common cocklebur, or *Xanthium strumarium*. (Figure 3).

## Discussion

Findings from our investigation suggest that this lethal outbreak (case fatality ratio 25%) was caused by toxic poisoning from *ghagra shak*, also known as *Xanthium strumarium*. In our epidemiologic investigation, consuming the plant was strongly and significantly associated with developing both vomiting and vomiting with unconsciousness. Thirty-one of 33 cases interviewed reportedly ate *ghagra shak* in the 24 hours preceding illness which provides further evidence that eating *X. strumarium* caused this outbreak. Our clinical findings are also consistent with the clinical description of a human case-series in Turkey associated with eating *X. strumarium* seeds where onset of symptoms began with nausea and vomiting and in some cases progressed quickly (within hours) to unconsciousness [7]. Liver enzymes were similarly elevated in patients who died in Turkey (ALT>7000 U/L) and in this outbreak (6795 and 10,000 U/L). In addition, our case fatality ratio was similar to the Turkish case series (33%) and the 3 deaths in Turkey occurred in children ≤10 years of age, which is the age group where most deaths occurred in this outbreak (13/19). Finally, *X. strumarium* is a biologically plausible cause of the outbreak as the plant's toxicity has been previously described. The toxic agent in the plant is carboxyatractyloside, which is present in the seeds and seedlings during the cotyledon stage but is absent in plants with 4 or more leaves [8]. A study of rats given an intraperitoneal LD50 dose of carboxyatractoloside suggests that the chemical's cytotoxic and lethal effects are likely due to its active metabolite. [9] Numerous outbreaks in livestock have been reported in association with consuming *X. strumarium* seedlings and seeds. [10]–[12]

The real culprit behind this outbreak, however, was food scarcity. Following the destructive floods in 2007 and high increases in the price of rice, food sources typically available to villagers in this area were scarce. Our respondents explained that because of this food shortage the villagers ate whatever was growing in their area, and thus, during November 2007 when *X. strumarium* plants began to sprout, they became a main meal. *X. strumarium* is regularly used in these communities in small amounts without incident so they had no reason to fear eating the plant. However, it appears that while eating small amounts was safe, consuming larger quantities was not. Although there are no specific toxic or lethal dose estimates published for *X. strumarium* seedlings in humans, when pigs were fed seedlings at .75-3% of body weight they developed illness and died [11]. If we apply this lethal dose to a child weighing 13 kg, the child would have to eat .98 kg of seedlings to receive a lethal dose. There are quite likely differences in the lethal dose between pigs and humans, but this calculation suggests that even in small amounts, consumption of the seedlings per se would not cause this kind of outbreak. Rather, it was the practice of consuming relatively larger amounts of this seedling as a meal that was to blame. As described to us by community members, their consumption practice changed during the outbreak period because of lack of other food sources. Although undernutrition is the most frequently cited result of food scarcity in low income countries, food scarcity also leads people to consume unsafe foods.

Cases resided in remote villages and others may have also fallen ill but not sought care; thus, some cases may remain unknown. Indeed, during our house-to-house survey in 2 villages we identified 11 new cases of vomiting with unconsciousness. Considering the worldwide distribution of this plant [13], its use as human food in this part of the world, the chronic problem of food insecurity in South Asia [14], and weak surveillance systems, cases likely occur but go unnoticed by local health officials. Follow-up studies to assess the true frequency of illness associated with consuming this plant and the burden of disease associated with consuming unsafe foods during times of food shortages should be considered.

In the epidemiological study only 46% of persons experiencing vomiting and unconsciousness, or their proxies, admitted to consuming *ghagra shak*. This finding conflicts with our qualitative data where many of the same individuals described their *ghagra shak* consumption to the study team and calls into question the role of this plant in causing the outbreak. However, we know that some villagers might have been unwilling to report preparing the plant to escape blame for causing deaths. Therefore, we believe that the anthropological findings on this topic are more reliable considering the in-depth nature of this methodology compared to the household surveys conducted for the exposure study. In addition, the anthropological data collection occurred during the outbreak while the exposure study began weeks later which may have affected recall of eating the plant. Despite this assumed under-reporting, the exposure to *ghagra shak* was strongly associated with illness and explained the largest portion of the cases in the outbreak.

This outbreak is further example of how poverty puts individuals at higher risk for disease and death. Public health messages advising against eating young *ghagra shak* should be developed and widely disseminated in Bangladesh and other areas of the sub-continent where the plants grow to prevent future outbreaks. Even during food shortages, people should be advised to not eat this plant. The issue of food insecurity, however, cannot be solved by public health communication. Although more than half of all childhood deaths in low income countries are associated with undernutrition [15], this alone does not represent the total burden of food insecurity. Assuring access to safe, healthy food is an urgent global priority, including in Bangladesh where an estimated 34 million people survive on less than 1805 calories a day [1].
